# The presence of a cryptic barrier in the West Pacific Ocean suggests the effect of glacial climate changes on a widespread sea‐dispersed plant, *Vigna marina* (Fabaceae)

**DOI:** 10.1002/ece3.5099

**Published:** 2019-07-04

**Authors:** Takashi Yamamoto, Yoshiaki Tsuda, Koji Takayama, Reiko Nagashima, Yoichi Tateishi, Tadashi Kajita

**Affiliations:** ^1^ Iriomote Station, Tropical Biosphere Research Center University of the Ryukyus Okinawa Japan; ^2^ United Graduate School of Agricultural Science Kagoshima University Kagoshima Japan; ^3^ Sugadaira Research Station, Mountain Science Center University of Tsukuba Ueda‐shi Japan; ^4^ Department of Botany, Graduate School of Science Kyoto University Kyoto Japan; ^5^ Museum of Natural and Environmental History Shizuoka Japan; ^6^ Department of Biology, Graduate School of Science Chiba University Chiba Japan; ^7^ Faculty of Education University of the Ryukyus Okinawa Japan

**Keywords:** approximate Bayesian computation, demographic history, long distance dispersal, pantropical plants with sea‐drifted seeds, phylogeography, refugia

## Abstract

Ocean currents are an important driver of evolution for sea‐dispersed plants, enabling them to maintain reciprocal gene flow via sea‐dispersed diaspores and obtain wide distribution ranges. Although geographic barriers are known to be the primary factors shaping present genetic structure of sea‐dispersed plants, cryptic barriers which form clear genetic structure within oceanic regions are poorly understood. To test the presence of a cryptic barrier, we conducted a phylogeographic study together with past demographic inference for a widespread sea‐dispersed plant, *Vigna marina*, using 308 individuals collected from the entire Indo‐West Pacific (IWP) region. Chloroplast DNA variation showed strong genetic structure that separated populations into three groups: North Pacific (NP), South Pacific (SP) and Indian Ocean (IN) (*F*′_CT_ among groups = 0.954–1.000). According to the Approximate Bayesian computation inference, splitting time between NP and SP was approximately 20,200 years (95%HPD, 4,530–95,400) before present. Moreover, a signal of recent population expansion was detected in the NP group. This study clearly showed the presence of a cryptic barrier in the West Pacific region of the distributional range of *V. marina*. The locations of the cryptic barrier observed in *V. marina* corresponded to the genetic breaks found in other plants, suggesting the presence of a common cryptic barrier for sea‐dispersed plants. Demographic inference suggested that genetic structure related to this cryptic barrier has been present since the last glacial maximum and may reflect patterns of past population expansion from refugia.

## 1 INTRODUCTION

Oceans are important corridors for estuarine and coastal plant species as they enable the exchange of individuals and help to maintain genetic uniformity with remote populations across distributional ranges. Many plant species growing in coastal environments produce diaspores (mainly seeds and fruits) specifically adapted to long distance dispersal (LDD, Cain, Milligan, & Strand, 2000) by ocean currents (Ridley, 1930). As oceanic dispersal (sea‐dispersal) is often the most effective mode of long distance seed dispersal (Harwell & Orth, 2002), many sea‐dispersed plants have extremely wide distributional ranges in the tropics and subtropics worldwide (Tomlinson, 1986). Thus, low genetic differentiation among populations could be expected due to their extended gene flow by LDD of diaspores across the distributional range (Kudoh & Whigham, 1997; Nilsson, Brown, Jansson, & Merritt, 2010). For example, genetic variation in not only maternally inherited chloroplast DNA (Takayama, Kajita, Murata, & Tateishi, 2006) but also bi‐parentally inherited nuclear DNA (Takayama, Tateishi, Murata, & Kajita, 2008) showed an absence of population differentiation between the Pacific and Indian Oceans in *Hibiscus tiliaceus*, suggesting high gene flow among populations and the absence of genetic barriers across their distribution. Alternatively, it has been suggested that LDD of sea‐dispersed plants is more limited than initially expected due to land and water barriers as well as the mobility and survival of diaspores during dispersal (Duke, Lo, & Sun, 2002; Triest, 2008). Indeed, clear genetic structure across land masses has been observed in certain mangroves (e.g. see a review by Triest, 2008). For example, recent studies clarified that the Malay Peninsula acts as a genetic barrier in *Bruguiera gymnorhiza* (Minobe et al., 2010; Urashi, Teshima, Minobe, Koizumi, & Inomata, 2013) and *Ceriops tagal* (Huang et al., 2012), and that the Central American Isthmus is a genetic barrier in *Hibiscus pernambucensis *and *Rhizophora mangle* (Takayama et al., 2006; Takayama, Tamura, Tateishi, Webb, & Kajita, 2013). Even when no clear land barrier exists, clear genetic structures were reported for mangrove species of *Rhizophora* (Takayama et al., 2013) as well as *Avicennia* (Mori, Zucchi, & Souza, 2015), in which the South Equatorial Current likely acted as a barrier to shape genetic structure in the Atlantic region.

Recent studies have reported genetic structure within oceanic regions where no apparent barriers exist, suggesting that inconspicuous barriers in the marine environment may exist (Duke et al., 2002). For example, Wee et al. (2015) detected clear genetic differentiation between South East Asian (Japan, Vietnam, Philippine and Indonesia) and Oceanian (Fiji, Vanuatu and New Caledonia) populations in *Rhizophora stylosa* and Guo et al. (2016) detected similar genetic structure in *Rhizophora apiculata*. As the locations of these genetic breaks might correspond to the boundaries of oceanic currents, Wee et al. (2015) suggested that oceanic circulation patterns might have acted as “cryptic barriers.” However, the presence of cryptic barriers has not yet been well examined. Firstly, previous studies (Guo et al., 2016; Wee et al., 2015) did not cover the entire species distributional range and the species studied by them are not widely distributed across the Indo‐West Pacific (IWP) region, which is one of the main biogeographical regions for mangroves. Extensive and detailed sampling is needed to test the presence of cryptic barriers, especially in the West Pacific region (sensu Wee et al., 2015). Additionally, extensive sampling may enable the evaluation of the strength of cryptic barriers in comparison to known geographical barriers (land barriers). For a deeper understanding of the process by which genetic structure related to cryptic barriers is formed, a comparative approach is required. Secondly, to understand cryptic barriers in detail, it is important to consider past population demography, such as past distribution changes, land barriers, LDD and ocean circulation, in addition to examining population genetic structure. Although the past demographic history of sea‐dispersed plants has not yet been well examined, recent advances in population genetics with approximate Bayesian computation (ABC) enable a deeper understanding of the past demographic dynamics of species, in terms of evolutionary parameters such as effective population size, time scale of divergence and population size change (Bertorelle, Benazzo, & Mona, 2010). By taking this approach we can deepen our understanding of the processes forming cryptic barriers.

In this study, we focused on a widespread coastal legume *Vigna marina* (Burm.) Merr. that grows in tropical and subtropical sandy beaches throughout the tropics (Verdcourt, 1971). The species is ranked as one of the most widely distributed flowering plants (Padulosi & Ng, 1993) and also a member of a group of “pantropical plants with sea‐drifted seeds” (Miryeganeh, Takayama, Tateishi, & Kajita, 2014; Takayama et al., 2006; Vatanparast, Takayama, Sousa, Tateishi, & Kajita, 2011). Some adaptations for oceanic dispersal are recognized in this species, such as remarkable salt tolerance (Tomooka, Kaga, Isemura, & Vaughan, 2011), seed buoyancy and viability in sea water (Nakanishi, 1988). Indeed, this species has a wider distributional range than any other tree species of mangroves (e.g. *B. gymnorhiza* and *R. stylosa*) in the IWP region. Therefore, this species can be a model species to test the presence of cryptic barriers across the IWP region. *Vigna marina* has a subspecies, *V. marina* ssp. *oblonga* (Benth.) Padulosi, but we did not use it in this study as it is only distributed in West Africa and has been suggested to be not so closely related (genetically) to *V. marina* (Sonnante, Spinosa, Marangi, & Pignone, 1997).

The aim of this study was to reveal the presence and process of formation of a cryptic barrier in *Vigna marina* in two ways: (a) to evaluate the genetic diversity and population genetic structure throughout the species distributional range using chloroplast DNA (cpDNA) and show if clear genetic structure caused by a cryptic barrier exists within the west Pacific region, and (b) to infer the past demographic history of *V. marina* in this region using the ABC approach to assess the processes of formation of the cryptic barrier that shaped the genetic structure.

## 2 MATERIALS AND METHODS

### 2.1 Plant sampling and DNA extraction

We sampled *Vigna marina* in thirty‐four localities and the sample size at each location ranged from one to 31 individuals, with a total sample size of 308 individuals (Supporting Information Table S1). To avoid sampling closely related individuals, each individual was selected randomly and separated from each other by at least 10 m. Collected leaf samples were dried by silica gel prior to DNA extraction. For the following data analyses, we treated some closely situated locations (< ca. 100 km) as a single location, resulting in a total of 21 locations (Table 1). Genomic DNA was extracted from dried leaf tissue using the cethyltrimethyl ammonium bromide (CTAB) based extraction method (Doyle & Doyle, 1987).

**Table 1:** Locality, sample size (*N*), and values of the genetic diversity parameters: numbers of observed haplotype (*S*), haplotype diversity (*H*) and nucleotide diversity (*π*) in 21 populations of *Vigna marina*

Oceanic region (SAMOVA group)	Code	Locality	Longitude	Latitude	*N*	*S*	*H*	*π* (×10^−3^)	Tajima's *D*	Fu and Li's *D*	Fu and Li's *F*
Pacific Ocean											
North Pacific (NP)					207	24	0.833	1.350	−0.963	−1.017	−1.197
	JA	Japan (Amami I. & Tokunoshima I.)	E129.086316	N27.973083	10	7	0.911	1.850	−0.163	−0.406	−0.389
	JO	Japan (Ogasawara Is.)	E142.142472	N26.698111	16	1	0.000	0.000	NA	NA	NA
	JR	Japan (Ryukyu I.)	E128.090947	N26.534842	31	5	0.729	2.040	2.523*	1.405*	2.049**
	JI	Japan (Iriomote I.)	E123.790643	N24.321466	6	4	0.800	1.840	−0.943	−0.913	−0.995
	TW	Taiwan	E120.876635	N22.009236	11	3	0.345	0.230	−0.778	−0.330	−0.494
	HW	U.S.A. (Hawaii Is.)	W158.204428	N21.581600	7	1	0.000	0.000	NA	NA	NA
	GU	U.S.A. (Guam I.)	E144.716608	N13.322893	26	3	0.532	0.980	0.538	1.282	1.236
	PH	Philippine	E122.225980	N11.794470	12	3	0.318	0.150	−1.451	−1.720	−1.865
	PA	Palau	E134.628875	N7.596514	14	5	0.703	0.790	−1.451	−1.720	−1.865
	MP	Micronesia (Pohnpei I.)	E158.205440	N6.976270	30	5	0.660	1.000	−0.079	0.204	0.137
	MK	Micronesia (Kosrae I.)	E163.020939	N5.346019	31	7	0.735	1.720	0.871	1.470*	1.504
	SA	Samoa	W171.45024	S14.04597	13	2	0.154	0.070	−1.149	−1.365	−1.481
South Pacific (SP)					77	5	0.296	0.970	−0.106	0.169	0.088
	AU	Australia	E145.466567	S16.088507	1	1	—	—	NA	NA	NA
	TH	French Polynesia (Tahiti I.)	W149.369021	S17.699975	34	2	0.371	1.510	1.560	1.364	1.669
	VU	Vanuatu	E168.443333	S17.816667	5	1	0.000	0.000	NA	NA	NA
	FJ	Fiji	E178.008467	S18.167592	12	2	0.167	0.680	−2.016**	−2.494**	−2.692**
	TO	Tonga	W175.17111	S21.256610	11	3	0.491	0.900	−2.011**	−2.428**	−2.627**
	NC	New Caledonia	E166.951461	S22.166780	14	1	0.000	0.000	NA	NA	NA
Indian Ocean											
Indian Ocean (IN)					24	1	0.000	0.000	NA	NA	NA
	MM	Myanmar	E95.310138	N15.961929	10	1	0.000	0.000	NA	NA	NA
	SC	Seychelles	E55.431250	S4.615111	11	1	0.000	0.000	NA	NA	NA
	MZ	Mozambique	E32.651420	S25.911630	3	1	0.000	0.000	NA	NA	NA
Total					308	29	0.864	1.870			

Note

**Figure 1:**
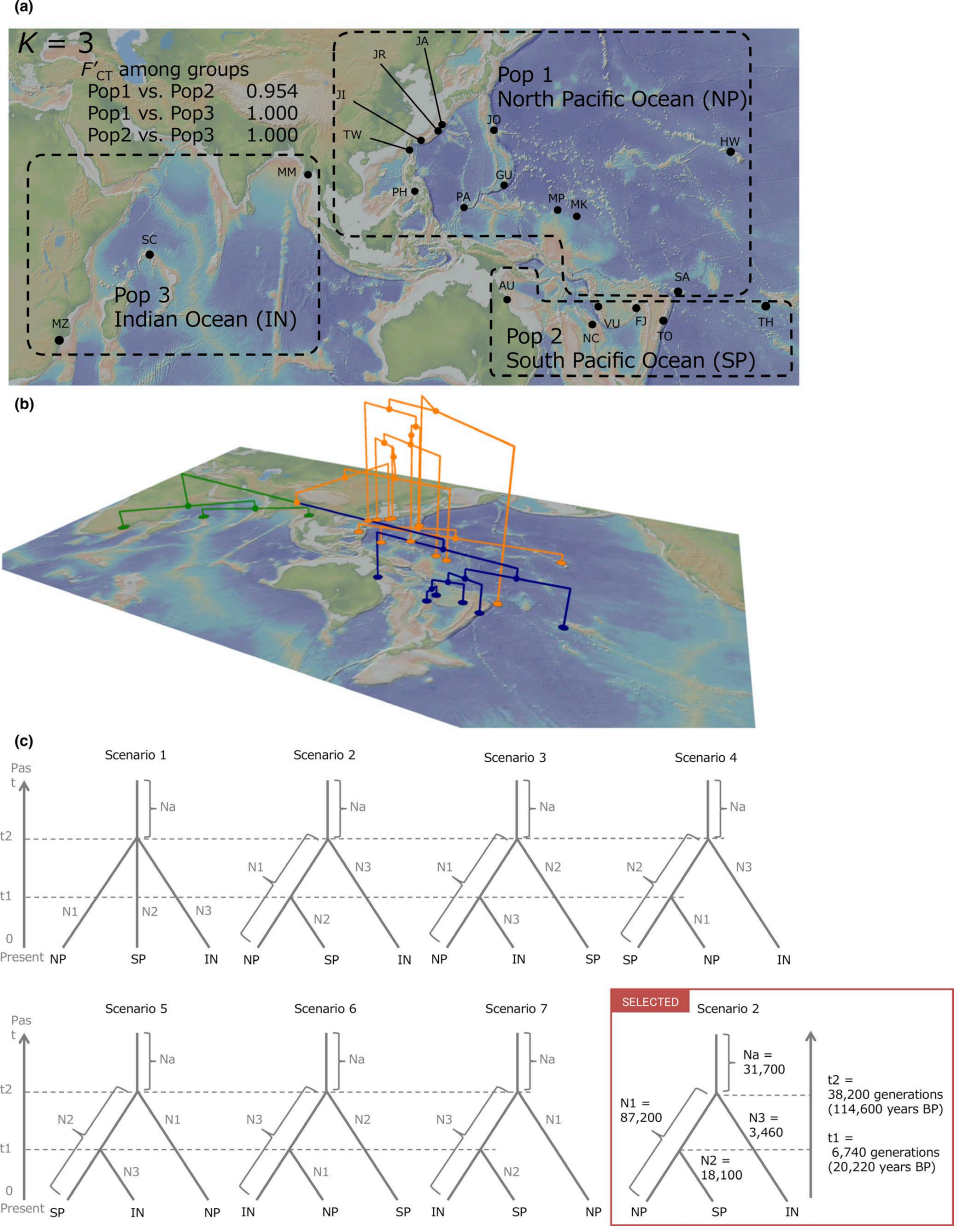
The definition of the three populations tested in *ABC1 *using DIYABC. (a) Results of the spatial analysis of molecular variance (SAMOVA) and pairwise *F*′_CT_ value among three groups. (b) The population Neighbor‐joining (NJ) tree reconstructed on a topographic map. Each branch was colored based on the results of SAMOVA (Pop1 = orange, Pop2 = blue, Pop3 = green). (c) The seven scenarios tested in *ABC1 *and the summary of the estimated parameters of the most‐likely scenario (= scenario 2). In all scenarios, t# represents time scale measured in number of generations and N# represents effective population size of the corresponding populations (Pop1, 2, 3, “a” before divergence) during the relevant time period (e.g. 0–t1, t1–t2)

Tajima's *D* (Tajima, 1989), and Fu and Li's *F* and *D* (Fu & Li, 1993) were calculated for populations with cpDNA variations (NA = not analyzed, *p < 0.10, **p < 0.02). We treated some closely located populations (<ca. 100 km) as a single population. Group is the one indicated by SAMOVA (see Figure 1a).

### 2.2 Molecular markers

PCR amplifications were performed using three cpDNA universal primer pairs; *trnT‐trnL* IGS (Taberlet, Gielly, Pautou, & Bouvet, 1991), *psbB‐psbH* IGS (Shinozaki et al., 1986) and *psbD‐trnT* IGS (Tun & Yamaguchi, 2008). TaKaRa *ExTaq* DNA Polymerase (TaKaRa Bio) and Mighty Amp^TM^ DNA Polymerase (TaKaRa Bio) were used for PCR. After purifying the PCR products using illustra ExoProStar (General Electric Company), the PCR products were sequenced directly using an ABI 3500 Genetic Analyzer (Applied Biosystems), an ABI 3130 xl Genetic Analyzer (Applied Biosystems) or a DNA sequence service supplied by Eurofins MWG operon company (Ebersberg, Germany). The DNA sequences were aligned using MUSCLE as implemented in MEGA ver. 6.0 (Tamura, Stecher, Peterson, Filipski, & Kumar, 2013) and manually edited to improve alignment. A total of 308 individuals were sequenced and representative cpDNA haplotype sequences of *V. marina* were registered in Genbank (accessions: LC270330–LC270363). For the following analyses, we removed all sequence gaps by using TRIMAI ver. 1.3 (Capella‐Gutiérrez, Silla‐Martínez, & Gabaldón, 2009) and used 2,212 bp in total.

### 2.3 Genetic diversity, phylogeny and population structure

The haplotype diversity (*H*), nucleotide diversity (*π*), Tajima's *D* (Tajima, 1989), and Fu and Li's *F* and *D* (Fu & Li, 1993) were calculated over all locations, for each of the three SAMOVA groups (see below) and for each location separately using the program DNASP ver. 5.10 (Librado & Rozas, 2009). The haplotype network was constructed and edited using the statistical parsimony procedure implemented in NETWORK ver. 4.613 (www.fluxus-engineering.com) following the median‐joining method (Bandelt, Forster, & Röhl, 1999). To evaluate population group structure, we used the spatial analysis of molecular variance (SAMOVA; Dupanloup, Schneider, & Excoffier, 2002) algorithm. SAMOVA is based on a simulated annealing procedure that aims to maximize the *F*_CT_ and the proportion of total genetic variance due to differences between groups of populations in an analysis of molecular variance (AMOVA), and to define groups of populations that are geographically homogeneous and maximally differentiated from each other. Here, the algorithm was performed by SAMOVA software (Dupanloup et al., 2002), which detected the number, *K*, of groups giving the largest *F*_CT_ value. The *K* was user‐defined and set between two and five with 500 independent simulated annealing processes in each run. Pairwise genetic differentiation described as *F*_CT _and its standardized value *F*'_CT_ (Meirmans & Hedrick, 2011) were calculated for all possible pairs of SAMOVA groups using GENALEX 6.5 (Peakall & Smouse, 2012). To evaluate genetic relationships among populations, we reconstructed a neighbor‐joining (NJ) tree based on genetic distance (*D*_A_) (Nei, Tajima, & Tateno, 1983) among populations and tested the statistical confidence of the NJ tree topology based on 1,000 bootstraps using Populations 1.2.30 beta software (Langella, 2007). The NJ tree was reconstructed on a topographic map using GeoMapApp (http://www.geomapapp.org/; Ryan et al., 2009) and GenGIS2 (Parks et al., 2009).

### 2.4 Inference past demographic history

The software DIYABC ver. 2.0 (Cornuet et al., 2008,2014) was used to determine population demography based on the approximate Bayesian computation (ABC) approach. We subdivided the analysis into “*Infer population demographic history”* (*ABC1*) and “*Infer population size change in the North Pacific group”* (*ABC2*). To simplify the scenarios in the ABC analysis, we defined three populations based on the result of SAMOVA: North Pacific group (NP), South Pacific group (SP) and Indian Ocean group (IN) (Figure 1a). In all scenarios, t# represents time scale measured in number of generations and N# represents effective population size of the corresponding populations (Pop1, 2, 3, “a” before divergence) during the relevant time period (e.g. 0–t1, t1–t2).

#### 2.4.1 ABC1*: Infer population demographic history*

To evaluate the demographic history of the three SAMOVA groups, we tested seven simple population divergence scenarios (Figure 1c).

The scenarios were as follows:

Scenario 1—Simple split model: all three populations merged at the same time, namely t2.
Scenario 2—Hierarchical split model: NP merged with SP at t1 and then with IN at t2.
Scenario 3—Hierarchical split model: NP merged with IN at t1 and then with SP at t2.
Scenario 4—Hierarchical split model: SP merged with NP at t1 and then with IN at t2.
Scenario 5—Hierarchical split model: SP merged with IN at t1 and then with NP at t2.
Scenario 6—Hierarchical split model: IN merged with NP at t1 and then with SP at t2.
Scenario 7—Hierarchical split model: IN merged with SP at t1 and then with NP at t2.

The default prior values were used for all parameters, including mutation rates, except for maximum values of effective population size (N1, N2, N3, Na) and maximum values of time scales (Supporting Information Table S2).

#### 2.4.2 ABC2*: Infer population size change in the North Pacific group*

**Figure 2:**
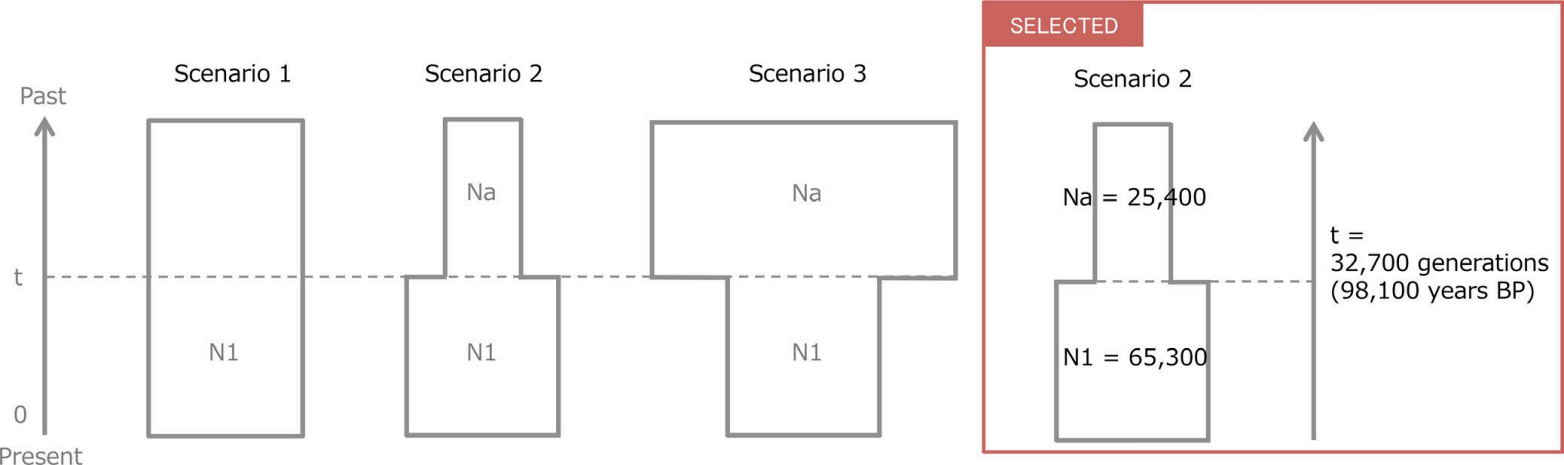
The three scenarios tested in *ABC2 *and the summary of the estimated parameters of the most‐likely scenario (= scenario 2). In all scenarios, t# represents time scale measured in number of generations and N# represents effective population size of the corresponding populations during the relevant time period

To examine the past transition of the effective population size of the North Pacific group (NP), which had enough haplotype diversity for within group demographic analysis, we tested three simple population demographic scenarios (Figure 2).

The scenarios were as follows:

Scenario 1—Constant model: effective population size of NP was constant at N1 from the present to the past. (Na = N1)
Scenario 2—Expansion model: effective population size of NP changed from Na to N1 at t. (Na < N1)
Scenario 3—Bottleneck model: effective population size of NP changed from Na to N1 at t. (Na > N1)

We changed the priors of the maximum population size and the maximum values of time scale from 10,000 (default value) to 100,000 to obtain better posterior distributions based on the results from the pilot runs (Supporting Information Table S2). The mutation model was selected using the program JMODELTEST ver. 2.0 (Darriba, Taboada, Doallo, & Posada, 2012; Guindon & Gascuel, 2003). The number of haplotypes, the number of segregating sites, the mean of pairwise differences, the variance of pairwise differences, Tajima's *D*, the number of private segregating sites and the mean of numbers of the rarest nucleotide at segregating sites were used as summary statistics for each of the three populations. The number of haplotypes, the number of segregating sites, the mean of pairwise differences and *F*_ST_ were used as the summary statistics for each of the population pairs. One million simulations were run for each scenario in both *ABC1* and *2*. After all the simulations had been run, the most‐likely scenario was determined by comparing the posterior probabilities using the logistic regression method. The goodness of fit of the scenario was assessed by the option ‘“model checking”’ with principal component analysis (PCA) in DIYABC, which measures the discrepancy between model and real data.

## 3 RESULTS

### 3.1 Genetic diversity and population structure

In total, 29 haplotypes were detected and high levels of genetic diversity (haplotype diversity, *H* = 0.864 and nucleotide diversity, *π* = 1.870 × 10^−3^) were observed throughout the species distributional range (Table 1). The haplotype diversity, *H*, ranged from 0.000 to 0.911 and the nucleotide diversity, *π*, ranged from 0.000 to 2.040 × 10^−3^. High values of haplotype and nucleotide diversity were mostly observed in the North‐West Pacific populations and in contrast, low diversities were mostly observed in the South Pacific and Indian Ocean populations. The Fiji and Tonga populations showed significantly negative values of Tajima's *D*, and Li and Fu's *D* and *F*, which (assuming the neutrality of examined regions of cpDNA) indicate recent population expansion in this region. In contrast, significantly positive values were observed in the Ryukyu population suggesting a recent bottleneck, possibly due to this population being located near to northern margin of the species distributional range. For the NP and SP groups, although no significant signal of population expansion or contraction was detected based on Tajima's *D*, and Li and Fu's *D* and *F* indexes, the ABC2 inference showed significant signal of population expansion in the NP group (see below).

**Figure 3:**
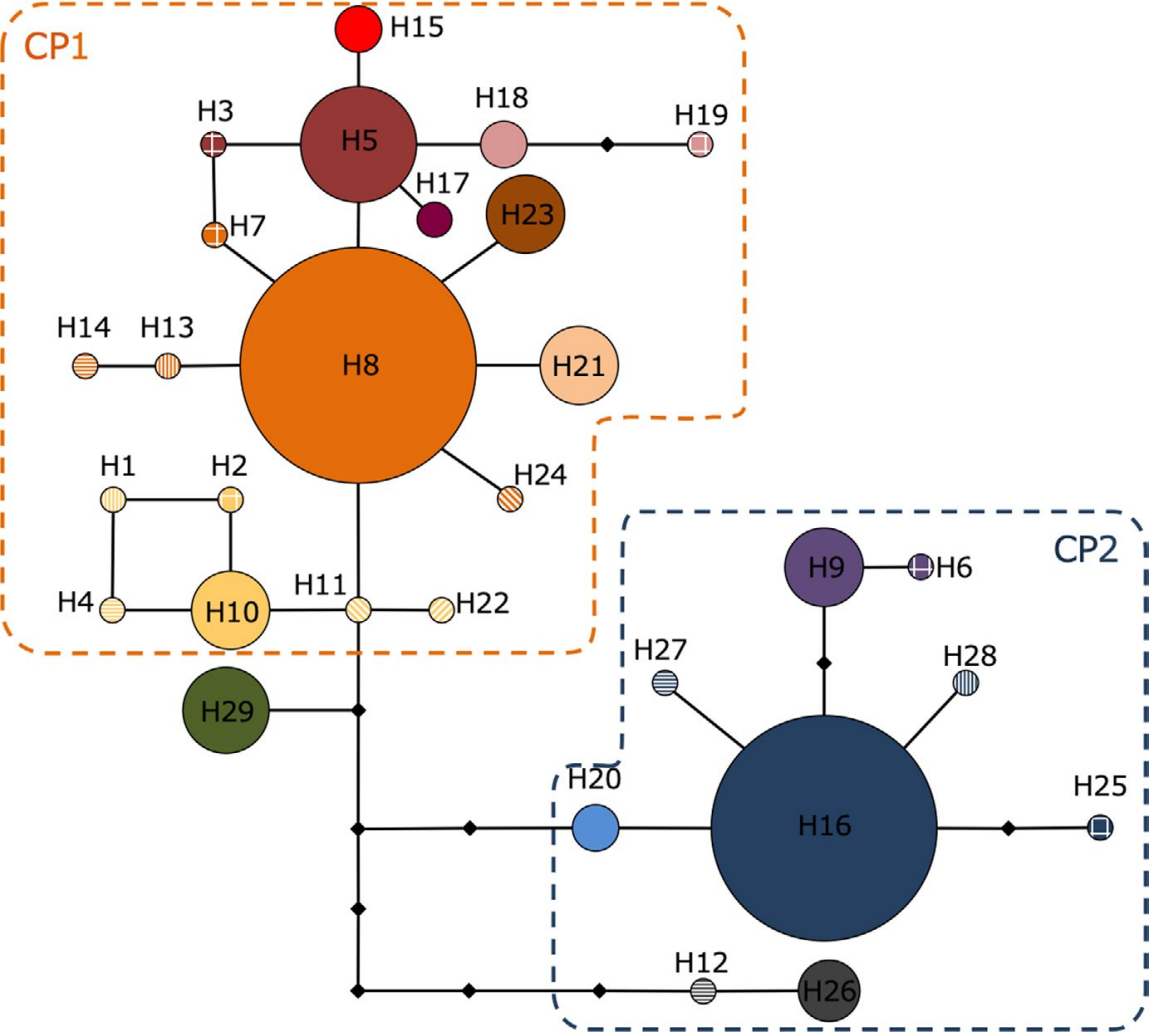
Median‐joining cpDNA haplotype network for *V. marina*. Each circle represents a single cpDNA haplotype and the size of the circle corresponds to the number of individuals

**Figure 4:**
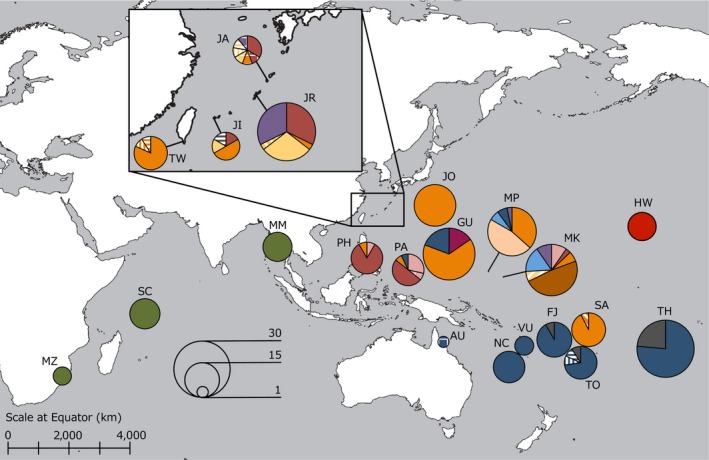
Geographic distribution of 29 cpDNA haplotypes. The size of the circle represents the sample size of the population

The median‐joining network showed the phylogenetic relationship among the 29 haplotypes and grouped them into at least two star‐shaped haplotype groups: CP1 and CP2 (Figure 3). These star‐shaped groups were composed of a few, frequently observed haplotypes (H8 and H16) at the center of networks and several, rare, local satellite haplotypes, suggesting recent population expansion (Avise, 2000). These group structures also reflected the geographic distribution of haplotypes (Figure 4). Most of the haplotypes belonging to CP1 were observed in the North Pacific populations and most of the haplotypes belonging CP2 were observed in the South Pacific region. Despite high levels of haplotype diversity being observed throughout the Pacific populations, all three Indian Ocean populations were fixed for only a single haplotype (H29) located just one step distance from the CP1 cluster.

The SAMOVA revealed the highest *F*_CT_ value (0.547; *p* < 0.01) when the 21 populations were divided into *K = *3 groups, composed of the North Pacific group (NP), South Pacific group (SP) and the Indian Ocean region (IN) (Figure 1a). The NJ tree also showed a similar pattern to the SAMOVA, revealing genetic differentiation among the three regions (Figure 1b, Supporting Information Figure S5). The pairwise *F*_CT_ values among the three regions ranged from 0.375 to 0.802 and their standardized values, described as *F*'_CT_, showed higher levels of genetic differentiation (0.954–1.000; Figure 1a).

### 3.2 Inference of past demographic history

In ABC1, the highest value of posterior probability was presumed for scenario 2 (0.5100, 95%HPD: 0.4832–0.5368) and the 95%HPD did not overlap with those of the other scenarios. For scenario 2, the median values of effective population size of N1 (North Pacific), N2 (South Pacific), N3 (Indian Ocean) and Na (putative ancestral population) were 81,400 (95%CI: 52,600 – 99,400), 23,400 (95%CI: 3,640.00–76,600), 6,010 (95%CI: 271–27,900), and 37,400 (95%CI: 1,230–94,600), respectively. The median value of t1, the divergence time when SP split from NP was 9,170 (95%CI: 1,510–31,800) generations ago and that of t2, the divergence time when IN split from NP and SP was 38,200 (95%CI: 8,510–93,700) generations ago. These time scales of t1 and t2 converted into absolute time as 20,200 and 114,600 years ago assuming a generation time for *V. marina* of three years according to our field observations. The median values of the mean mutation rate of cpDNA and the median values of mean coefficient k C/T were estimated as 2.92 × 10^−8^ (95%CI: 1.51 × 10^−8^–5.50 × 10^−8^) and 11.50 (95%CI: 8.15 × 10^−1^–2.00 × 10^1^), respectively (Supporting Information Figure S1 and Table S3). The estimated values of 39 summary statistics did not show significant differences between the observed and simulated data based on the posterior distributions (Supporting Information Table S4). The PCA showed that the observed data were located around the center of the cluster of points of the simulated data based on the posterior distributions (Supporting Information Figure S2), suggesting that scenario 2 was a good fit for the observed data.

In ABC2, the highest value of posterior probability was presumed for scenario 2 (population expansion model; 0.4766, 95%HPD: 0.4708–0.4824) and its posterior probability did not overlap and was much higher than that for scenario 1 (constant population size model; PP = 0.2928, 95%CI: 0.2875–0.2981) and scenario 3 (bottleneck model; PP = 0.2306, 95%CI: 0.2257–0.2355) (Table 2). The median values of the effective population size of N1 (current populations of North Pacific) and Na (putative ancestral population) were 64,900 (95%CI: 26,900–98,400) and 29,800 (95%CI: 1,290–79,900), respectively. The median value of t, time scale when population expansion occurred, was 40,200 (95%CI: 2,320–96,500) generations ago, which converted into absolute time as 98,100 years ago. The median values of the mean mutation rate of cpDNA and the median values of mean coefficient *k* C/T were estimated as 4.96 × 10^−8^ (95%CI: 2.32 × 10^−8^–9.39 × 10^−8^) and 11.40 (95%CI: 7.81 × 10^−1^–1.99 × 10^1^), respectively (Supporting Information Figure S3 and Table S3). The estimated values of eight summary statistics did not show significant differences between the observed and simulated data based on the posterior distributions (Supporting Information Table S4). The PCA suggested that scenario 2 was a good fit for the observed data (Supporting Information Figure S4).

**Table 2:** Posterior probability of each scenario and its 95% confidence interval based on the logistic estimate by DIYABC

Scenario	Posterior probability	95% CI (lower–upper)
ABC1		
1	0.0681	0.0000–0.1528
2	0.5100	0.4832–0.5368
3	0.0137	0.0000–0.0864
4	0.3861	0.3609–0.4114
5	0.0109	0.0000–0.0838
6	0.0076	0.0000–0.0809
7	0.0035	0.0000–0.0772
ABC2		
1	0.2928	0.2875–0.2981
2	0.4766	0.4708–0.4824
3	0.2306	0.2257–0.2355

## 4 DISCUSSION

### 4.1 Presence of a cryptic barrier in the West Pacific

This study clearly showed strong genetic structure within the distributional range of the widespread sea‐dispersed plant *V. marina*. The clear genetic break observed in the West Pacific where no geographic barrier exists suggests the presence of a cryptic barrier that prevents seed‐mediated gene flow. The genetic clustering analyses (SAMOVA and population NJ tree) showed clear genetic structure that divides the distributional range of *V. marina* into three regional clusters: North Pacific (NP), South Pacific (SP) and Indian Ocean (IN) (Figures 1a,b and 4). The haplotype composition of the NP and SP regions were almost completely different and *F'*_CT_ values among all three regions were high (0.954–1.000), suggesting very limited gene flow among regions. In addition, potential patterns of admixture between the NP and SP regions were found only in the populations located near to the boundary of the two genetic clusters. Finally, the median‐joining network showed that the two major groups of haplotypes, CP1 (NP) and CP2 (SP), were phylogenetically differentiated from each other. On the other hand, most of the major haplotypes were highly shared among populations within each of the NP or SP clusters, suggesting substantial gene flow among populations within each genetic cluster. In addition, the AMOVA suggested only a low proportion of variation was due to differences among populations within clusters (9.17%; Table 3). These results imply that despite *V. marina* having the ability for LDD, gene flow between the NP and SP regions has been historically prevented even though no geographical barrier exists.

**Table 3:** Results of the analysis of molecular variance (AMOVA) performed considering the three cpDNA population groups defined in the spatial analysis of molecular variance (SAMOVA; Dupanloup et al., 2002)

Source of variation	*df*	Sum of squares	Variance components	Percentage of variation
Among groups	2	246.799	1.598	54.73
Among populations within groups	18	87.215	0.268	9.17
Within populations	287	302.506	1.054	36.10
Total	307	636.519	2.912	

Interestingly, the level of genetic differentiation in *V. marina* across the West Pacific cryptic barrier is as strong as across the Malay Peninsula region (*F'*_CT_ = 0.954 to 1.000; Figure 1a). This highlights that cryptic barriers should be recognized as just as important as other geographic barriers when evaluating the phylogeographic history and genetic diversity of *V. marina*. Generally, the Malay Peninsula is one of the most important present and historical geographical barriers (Duke et al., 2002; Voris, 2000) forming current patterns of phylogeographic structure and genetic diversity in several mangroves (Duke et al., 2002; Huang et al., 2012; Urashi et al., 2013) and other marine species (Alfaro, Karns, Voris, Abernathy, & Sellins, 2004; Gaither et al., 2011) distributed in the Indo‐West Pacific (IWP) region. The current data suggests that in addition to the well‐recognized geographic barrier of the Malay Peninsula, the cryptic barrier in the West Pacific has also strongly affected the phylogeographic structure and genetic diversity of the widespread sea‐dispersed plant *V. marina*.

A clear genetic break in the West Pacific Ocean has also been observed in some other sea‐dispersed plants: *Rhizophora stylosa* (Wee et al., 2015), *R. apiculata* (Guo et al., 2016), *Xylocarpus granatum* (Tomizawa et al., 2017) and *Sonneratia alba* (Wee et al., 2017), and in these cases the locations of the genetic breaks roughly corresponded to the one determined for *V. marina*. The presence of common genetic structure in multiple widespread sea‐dispersed species indicates the presence of a common cryptic barrier. Generally, widespread sea‐dispersed plants, including mangroves and “pantropical plants with sea‐drifted seeds” (Takayama et al., 2006), often have common genetic structures because they usually share some ecological features (such as habitat, species distribution, seed dispersal strategy) and phylogeographic history. These species are also likely to share common divergence histories relating to geographic and cryptic barriers. In this study, the ABC approach was employed to elucidate the historical processes that formed the clear genetic structure related to the West Pacific cryptic barrier observed in *V. marina*. Although these inferences merely represent the population demographic history relating to *V. marina*, they may also reflect common historical processes shared by multiple widespread sea‐dispersed plants distributed in the IWP region.

### 4.2 Formation of a cryptic barrier in *V. marina*

The demographic inference estimated that the splitting time between the NP and SP was 20,200 years (6,740 generations) before present (BP), corresponding to the period of the Last Glacial Maximum (21,000 years BP). This suggests that a cryptic barrier for *V. marina* was already present during the last glacial period. During the Quaternary period, glacial climate changes had drastic and complex effects on the distribution of tropical coastal species, for example, modifying the geography of coastlines and ocean current patterns, changing habitats, and inducing the contraction of species distributional ranges. Indeed, drastic sea level drop (~120 m in the Last Glacial Maxima: LGM) and subsequent landmass formation over large areas have been reported in South East Asia (Sunda shelf) and the Oceania region (Sahul shelf) (Voris, 2000). These landmass formations would have separated the distributional range of sea‐dispersed plants both directly and indirectly (via the modification of ocean currents), resulting in the restriction of seed‐mediated gene flow and population differentiation. Additionally, historical adaptation to multiple environments could also explain the observed population subdivision. Although it is difficult to elucidate which factor(s) formed the clear genetic break in the West Pacific distributional range of sea‐dispersed plants, this study suggests that genetic structure relating to the cryptic barrier has been maintained since the LGM.

A signal of recent population expansion was detected in the North Pacific cluster by ABC, and in two populations (Fiji and Tonga) in the South Pacific based on Tajima's *D* and Fu and Li's *D* and *F*, suggesting past population expansion of *V. marina* from glacial refugia after the LGM. Cannon, Morley, and Bush (2009) recently reported that the major refugia of lowland coastal plants and mangroves was in the South China Sea during the LGM period. It is well kwon that richness of alleles or haplotypes is expected to be higher in refugial areas than areas recolonized after the glacial period (Comps, Gömöry, Letouzey, Thiébaut, & Petit, 2001; Petit et al., 2003). Indeed, North Pacific populations (especially in South East Asia) of *Vigna marina* had much higher haplotype richness compared to other populations, implying that the genetic structure observed in *V. marina* may reflect the geographic patterns of refugia that existed during the LGM. Taking this information into account, we have an idea about the phylogeographic process of wide spread sea‐dispersed plants. Although LDD by sea‐dispersed seeds connected populations within the distribution range, contraction of the species distribution during the LGM caused population subdivision into at least two regions, possibly the South China Sea and the South Pacific Ocean, resulting in some fixed haplotype differences in each region. After the last glacial period, the South China Sea population expanded into the South Pacific Ocean, and may have had secondary contact with South Pacific haplotypes in the Oceania region. Indeed, the Samoa population was solely assigned to the NP group, unlike its neighboring populations, and multiple NP populations have CP2 haplotypes, which indicate recent LDD events between the NP and SP groups.

As this study demonstrates, the present phylogeographic structure of widespread sea‐dispersed plants has been more strongly influenced by glacial climate change than previously expected. For many widespread forest plants in temperate regions, glacial climate change is one of the key factors that shaped genetic structure via contraction to glacial refugia at the LGM and subsequent population expansion (reviewed in Hewitt, 2000; Provan & Bennett, 2008). For widespread sea‐dispersed plants in tropical regions, the influence of glacial climate change has been less expected, due to their extremely high ability for LDD that may easily homogenize populations by sea‐dispersed seeds, and also because the locations of refugia have not been clear for tropical plants. The findings of this study, as well as other recent ones using molecular markers and the ABC approach, provided evidence suggesting the presence of cryptic barriers in the West Pacific (for example, *Xylocarpus granatum *(Tomizawa et al., 2017); *Sonneratia alba* (Wee et al., 2017)) in relation to the LGM. These findings suggest that not only the widespread temperate forest plants, but also the sea‐dispersed tropical plants have been influenced by glacial climate change. By studying other sea‐dispersal plants in the same way, and also by using higher resolution markers such as multi‐locus nuclear DNA, we may obtain further support for this idea.

In this study, ABC analyses explored the population demographic history in relation to refugia during LGM or the past ice age previous to the LGM. Although statistical uncertainty remains (e.g., time scale, generation time, broad 95% CI range and assumed model, reviewed in detail see Tsuda, Nakao, Ide, & Tsumura, 2015), the ABC inferences used in this study were useful to explore the processes by which the cryptic barrier in widespread sea‐dispersed plants is formed. Regarding the model assumed in the ABC, DIYABC does not consider gene flow after divergence, and it may bias the inferred temporal parameters (Tsuda et al., 2015). However, as discussed previously, genetic differentiation among the examined regions was quite high (*F′*_CT_ = 0.954 to 1.000), suggesting highly restricted gene flow among them. Thus, as far as the demographic history among the three regional groups is concerned, the bias in this study is likely limited. In addition, as gene flow after divergence wasn't considered, divergence times might have been underestimated (Tsuda et al., 2015). However, the main discussion here would not be changed, with the population split still occurring before the LGM.

## 5 CONCLUSION

In this study, the presence of a cryptic barrier in the West Pacific distributional range of a widespread sea‐dispersed plant, *V. marina*, was clearly detected. The locations of the cryptic barrier observed in *V. marina* corresponded to the genetic breaks found in other plants, suggesting the presence of a common cryptic barrier for sea‐dispersed plants in the West Pacific region. Demographic inferences using the ABC approach suggested that genetic structure related to the cryptic barrier has been present since the last glacial period and may reflect patterns of past population expansion from refugia. These results suggest that despite high LDD ability that can homogenize genetic structure, present phylogeographic patterns of sea‐dispersed plants have been strongly affected by glacial climate changes, as reported in other land plants.

## CONFLICT OF INTEREST

None declared.

## AUTHOR CONTRIBUTIONS

T.Y., Y.Tsuda, K.T. and T.K. designed the study. T.Y., K.T., R.N., Y.Tateishi and T.K. carried out field collection. T.Y. and R.N. carried out the molecular work. T.Y., Y.Tsuda and R.N. analyzed the data. All authors contributed to the writing of the paper and all have approved of the final version.

## Data Availability

DNA sequences: Genbank accessions LC270330–LC270363. DNA alignment and input data for all analyses used in this study will be uploaded to Dryad. Data available from the Dryad Digital Repository: https://doi.org/10.5061/dryad.cv66pm4.

## References

[ece35099-bib-0001] Alfaro, M. E. , Karns, D. R. , Voris, H. K. , Abernathy, E. , & Sellins, S. L. (2004). Phylogeny of *Cerberus* (Serpentes: Homalopsinae) and phylogeography of *Cerberus rynchops*: Diversification of a coastal marine snake in Southeast Asia. Journal of Biogeography, 31, 1277–1292. 10.1111/j.1365-2699.2004.01114.x

[ece35099-bib-0002] Avise, J. C. (2000). Phylogeography: The history and formation of species (p. 464). Cambridge, MA: Harvard University Press.

[ece35099-bib-0003] Bandelt, H. J. , Forster, P. , & Röhl, A. (1999). Median‐joining networks for inferring intraspecific phylogenies. Molecular Biology and Evolution, 16, 37–48. 10.1093/oxfordjournals.molbev.a026036 10331250

[ece35099-bib-0004] Bertorelle, G. , Benazzo, A. , & Mona, S. (2010). ABC as a flexible framework to estimate demography over space and time: Some cons, many pros. Molecular Ecology, 19, 2609–2625. 10.1111/j.1365-294X.2010.04690.x 20561199

[ece35099-bib-0005] Cain, M. L. , Milligan, B. G. , & Strand, A. E. (2000). Long‐distance seed dispersal in plant populations. American Journal of Botany, 87, 1217–1227. 10.2307/2656714 10991892

[ece35099-bib-0006] Cannon, C. H. , Morley, R. J. , & Bush, A. B. G. (2009). The current refugial rainforests of Sundaland are unrepresentative of their biogeographic past and highly vulnerable to disturbance. Proceedings of the National Academy of Sciences of the United States of America, 106, 11188–11193. 10.1073/pnas.0809865106 19549829 PMC2708749

[ece35099-bib-0007] Capella‐Gutiérrez, S. , Silla‐Martínez, J. M. , & Gabaldón, T. (2009). trimAl: A tool for automated alignment trimming in large‐scale phylogenetic analyses. Bioinformatics, 25, 1972–1973. 10.1093/bioinformatics/btp348 19505945 PMC2712344

[ece35099-bib-0009] Comps, B. , Gömöry, D. , Letouzey, J. , Thiébaut, B. , & Petit, R. J. (2001). Diverging trends between heterozygosity and allelic richness during postglacial colonization in European beech. Genetics, 157, 389–397. 11139519 10.1093/genetics/157.1.389 PMC1461495

[ece35099-bib-0010] Cornuet, J.‐M. , Pudlo, P. , Veyssier, J. , Dehne‐Garcia, A. , Gautier, M. , Leblois, R. , … Estoup, A. (2014). DIYABC v2. 0: a software to make approximate Bayesian computation inferences about population history using single nucleotide polymorphism, DNA sequence and microsatellite data. Bioinformatics, 30, 1187–1189. 24389659 10.1093/bioinformatics/btt763

[ece35099-bib-0011] Cornuet, J.‐M. , Santos, F. , Beaumont, M. A. , Robert, C. P. , Marin, J.‐M. , Balding, D. J. , … Estoup, A. (2008). Inferring population history with DIY ABC: A user‐friendly approach to approximate Bayesian computation. Bioinformatics, 24, 2713–2719. 10.1093/bioinformatics/btn514 18842597 PMC2639274

[ece35099-bib-0012] Darriba, D. , Taboada, G. L. , Doallo, R. , & Posada, D. (2012). jModelTest 2: More models, new heuristics and parallel computing. Nature Methods, 9, 772 10.1038/nmeth.2109 PMC4594756 22847109

[ece35099-bib-0014] Doyle, J. J. , & Doyle, J. L. (1987). A rapid DNA isolation procedure for small quantities of fresh leaf tissue. Phytochemical Bulletin, 19, 11–15.

[ece35099-bib-0015] Duke, N. C. , Lo, Y. , & Sun, M. (2002). Global distribution and genetic discontinuities of mangroves – emerging patterns in the evolution of *Rhizophora* . Trees, 16, 65–79. 10.1007/s00468-001-0141-7

[ece35099-bib-0016] Dupanloup, I. , Schneider, S. , & Excoffier, L. (2002). A simulated annealing approach to define the genetic structure of populations. Molecular Ecology, 11, 2571–2581. 10.1046/j.1365-294X.2002.01650.x 12453240

[ece35099-bib-0018] Fu, Y. X. , & Li, W. H. (1993). Statistical tests of neutrality of mutations. Genetics, 133, 693–709. 8454210 10.1093/genetics/133.3.693 PMC1205353

[ece35099-bib-0019] Gaither, M. R. , Bowen, B. W. , Bordenave, T.‐R. , Rocha, L. A. , Newman, S. J. , Gomez, J. A. , … Craig, M. T. (2011). Phylogeography of the reef fish *Cephalopholis* *argus* (Epinephelidae) indicates Pleistocene isolation across the Indo‐Pacific Barrier with contemporary overlap in The Coral Triangle. BMC Evolutionary Biology, 11, 189 10.1186/1471-2148-11-189 21722383 PMC3145601

[ece35099-bib-0020] Guindon, S. , & Gascuel, O. (2003). A simple, fast, and accurate algorithm to estimate large phylogenies by maximum likelihood. Systematic Biology, 52, 696–704. 10.1080/10635150390235520 14530136

[ece35099-bib-0021] Guo, Z. , Huang, Y. , Chen, Y. , Duke, N. C. , Zhong, C. , & Shi, S. (2016). Genetic discontinuities in a dominant mangrove *Rhizophora apiculata* (Rhizophoraceae) in the Indo‐Malesian region. Journal of Biogeography, 43, 1856–1868.

[ece35099-bib-0022] Harwell, M. C. , & Orth, R. J. (2002). Long‐distance dispersal potential in a marine macrophyte. Ecology, 83, 3319–3330. 10.1890/0012-9658(2002)083[3319:LDDPIA]2.0.CO;2

[ece35099-bib-0023] Hewitt, G. (2000). The genetic legacy of the Quaternary ice ages. Nature, 405, 907–913. 10.1038/35016000 10879524

[ece35099-bib-0024] Huang, Y. , Zhu, C. , Li, X. , Li, X. , Hu, L. , Tan, F. , … Shi, S. (2012). Differentiated population structure of a genetically depauperate mangrove species *Ceriops* *tagal* revealed by both Sanger and deep sequencing. Aquatic Botany, 101, 46–54. 10.1016/j.aquabot.2012.04.001

[ece35099-bib-0025] Kudoh, H. , & Whigham, D. F. (1997). Microgeographic genetic structure and gene flow in *Hibiscus* *moscheutos* (Malvaceae) populations. American Journal of Botany, 84, 1285–1293. 21708685

[ece35099-bib-0026] Langella, O. (2007). Populations 1.2. 30: population genetic software (individuals or populations distances, phylogenetic trees). France. Retrieved from http://bioinformatics.org/populations/

[ece35099-bib-0027] Librado, P. , & Rozas, J. (2009). DnaSP v5: A software for comprehensive analysis of DNA polymorphism data. Bioinformatics, 25, 1451–1452. 10.1093/bioinformatics/btp187 19346325

[ece35099-bib-0028] Meirmans, P. G. , & Hedrick, P. W. (2011). Assessing population structure: FST and related measures. Molecular Ecology Resources, 11, 5–18. 10.1111/j.1755-0998.2010.02927.x 21429096

[ece35099-bib-0029] Minobe, S. , Fukui, S. , Saiki, R. , Kajita, T. , Changtragoon, S. , Ab Shukor, N. A. , … Yamazaki, T. (2010). Highly differentiated population structure of a Mangrove species, *Bruguiera* *gymnorhiza* (Rhizophoraceae) revealed by one nuclear GapCp and one chloroplast intergenic spacer trnF‐trnL. Conservation Genetics, 11, 301–310. 10.1007/s10592-009-9806-3

[ece35099-bib-0030] Miryeganeh, M. , Takayama, K. , Tateishi, Y. , & Kajita, T. (2014). Long‐distance dispersal by sea‐drifted seeds has maintained the global distribution of *Ipomoea* *pe*s‐*caprae* subsp. *brasiliensis* (Convolvulaceae). PLoS ONE, 9, e91836 10.1371/journal.pone.0091836 24755614 PMC3995641

[ece35099-bib-0031] Mori, G. M. , Zucchi, M. I. , & Souza, A. P. (2015). Multiple‐geographic‐scale genetic structure of two mangrove tree species : The roles of mating system, hybridization, limited dispersal and extrinsic factors. PLOS ONE, 10(2), 1–23. 10.1371/journal.pone.0118710 PMC4344226 25723532

[ece35099-bib-0032] Nakanishi, H. (1988). Dispersal ecology of the maritime plants in the Ryukyu. Ecological Research, 3, 163–173.

[ece35099-bib-0033] Nei, M. , Tajima, F. , & Tateno, Y. (1983). Accuracy of estimated phylogenetic trees from molecular data. Journal of Molecular Evolution, 19, 153–170. 10.1007/BF02300753 6571220

[ece35099-bib-0034] Nilsson, C. , Brown, R. L. , Jansson, R. , & Merritt, D. M. (2010). The role of hydrochory in structuring riparian and wetland vegetation. Biological Reviews, 85, 837–858. 10.1111/j.1469-185X.2010.00129.x 20233190

[ece35099-bib-0035] Padulosi, S. , & Ng, N. Q. (1993). A useful and unexploited herb, *Vigna marina *(Leguminosae ‐ Papilionoideae) and the taxonomic revision of its genetic diversity. Bulletin Du Jardin Botanique National De Belgique., 62, 119–126. 10.2307/3668270

[ece35099-bib-0036] Parks, D. H. , Porter, M. , Churcher, S. , Wang, S. , Blouin, C. , Whalley, J. , … Beiko, R. G. (2009). GenGIS: A geospatial information system for genomic data. Genome Research, 19, 1896–1904. 10.1101/gr.095612.109 19635847 PMC2765287

[ece35099-bib-0037] Peakall, R. , & Smouse, P. (2012). GenAlEx 6.5: Genetic analysis in Excel. Population genetic soft‐ware for teaching and research – An update. Bioinformatics, 1, 6–8. 10.1093/bioinformatics/bts460 PMC3463245 22820204

[ece35099-bib-0038] Petit, R. J. , Aguinagalde, I. , de Beaulieu, J. L. , Bittkau, C. , Brewer, S. , Cheddadi, R. , … Vendramin, G. G. (2003). Glacial refugia: Hotspots but not melting pots of genetic diversity. Science, 300, 1563–1565. 10.1126/science.1083264 12791991

[ece35099-bib-0039] Provan, J. , & Bennett, K. D. (2008). Phylogeographic insights into cryptic glacial refugia. Trends in Ecology and Evolution, 23, 564–571. 10.1016/j.tree.2008.06.010 18722689

[ece35099-bib-0040] Ridley, H. N. (1930). The dispersal of plants throughout the world (p. 744). Ashford, UK: L. Reeve & Co., Ltd.

[ece35099-bib-0041] Ryan, W. B. F. , Carbotte, S. M. , Coplan, J. O. , O'Hara, S. , Melkonian, A. , Arko, R. , … Zemsky, R. (2009). Global multi‐resolution topography synthesis. Geochemistry, Geophysics, Geosystems, 10(3). 10.1029/2008GC002332

[ece35099-bib-0042] Shinozaki, K. , Ohme, M. , Tanaka, M. , Wakasugi, T. , Hayshida, N. , Matsubayasha, T. , … Sugiura, M. (1986). The complete nucleotide sequence of tobacco chloroplast genome. Plant Molecular Biology Reporter, 4, 111–148.

[ece35099-bib-0043] Sonnante, G. , Spinosa, A. , Marangi, A. , & Pignone, D. (1997). Isozyme and RAPD analysis of the genetic diversity within and between *Vigna luteola* and *V. marina* . Annals of Botany, 80, 741–746. 10.1006/anbo.1997.0511

[ece35099-bib-0044] Taberlet, P. , Gielly, L. , Pautou, G. , & Bouvet, J. (1991). Universal primers for amplification of three non‐coding regions of chloroplast DNA. Plant Molecular Biology, 17, 1105–1109. 10.1007/BF00037152 1932684

[ece35099-bib-0045] Tajima, F. (1989). Statistical method for testing the neutral mutation hypothesis by DNA polymorphism. Genetics, 123, 585–595. 2513255 10.1093/genetics/123.3.585 PMC1203831

[ece35099-bib-0046] Takayama, K. , Kajita, T. , Murata, J. , & Tateishi, Y. (2006). Phylogeography and genetic structure of *Hibiscus* *tiliaceus* ‐ Speciation of a pantropical plant with sea‐drifted seeds. Molecular Ecology, 15, 2871–2881. 10.1111/j.1365-294X.2006.02963.x 16911207

[ece35099-bib-0047] Takayama, K. , Tamura, M. , Tateishi, Y. , Webb, E. L. , & Kajita, T. (2013). Strong genetic structure over the American continents and transoceanic dispersal in the mangrove genus *Rhizophora* (Rhizophoraceae) revealed by broad‐scale nuclear and chloroplast DNA analysis. American Journal of Botany, 100, 1191–1201. 23711904 10.3732/ajb.1200567

[ece35099-bib-0048] Takayama, K. , Tateishi, Y. , Murata, J. , & Kajita, T. (2008). Gene flow and population subdivision in a pantropical plant with sea‐drifted seeds *Hibiscus* *tiliaceus* and its allied species: Evidence from microsatellite analyses. Molecular Ecology, 17, 2730–2742. 18482261 10.1111/j.1365-294X.2008.03799.x

[ece35099-bib-0049] Tamura, K. , Stecher, G. , Peterson, D. , Filipski, A. , & Kumar, S. (2013). MEGA6: Molecular evolutionary genetics analysis version 6.0. Molecular Biology and Evolution, 30, 2725–2729. 10.1093/molbev/mst197 24132122 PMC3840312

[ece35099-bib-0050] Tomizawa, Y. , Tsuda, Y. , Saleh, M. N. , Wee, A. K. S. , Takayama, K. , Yamamoto, T. , … Kajita, T. (2017). Genetic structure and population demographic history of a widespread mangrove plant *Xylocarpus granatum* J. Koenig across the Indo‐West Pacific region. Forests, 8, 480.

[ece35099-bib-0051] Tomlinson, P. B. (1986). The botany of mangroves (p. 413). Cambridge, UK: Cambridge University Press.

[ece35099-bib-0052] Tomooka, N. , Kaga, A. , Isemura, T. , & Vaughan, D. (2011). Vigna In Kole C. (Ed.), Wild crop relatives: Genomic and breeding resources, Legume crops and forages (pp. 291–311). Berlin, Germany: Springer.

[ece35099-bib-0053] Triest, L. (2008). Molecular ecology and biogeography of mangrove trees towards conceptual insights on gene flow and barriers: A review. Aquatic Botany, 89, 138–154. 10.1016/j.aquabot.2007.12.013

[ece35099-bib-0054] Tsuda, Y. , Nakao, K. , Ide, Y. , & Tsumura, Y. (2015). The population demography of *Betula* *maximowicziana*, a cool‐temperate tree species in Japan, in relation to the last glacial period: Its admixture‐like genetic structure is the result of simple population splitting not admixing. Molecular Ecology, 24, 1403–1418. 25706115 10.1111/mec.13123

[ece35099-bib-0055] Tun, Y. T. , & Yamaguchi, H. (2008). Sequence variation of four chloroplast non‐coding regions among wild, weedy and cultivated *Vigna* *angularis* accessions. Breeding Science, 58, 325–330. 10.1270/jsbbs.58.325

[ece35099-bib-0056] Urashi, C. , Teshima, K. M. , Minobe, S. , Koizumi, O. , & Inomata, N. (2013). Inferences of evolutionary history of a widely distributed mangrove species, *Bruguiera* *gymnorrhiza*, in the Indo‐West Pacific region. Ecology and Evolution, 3, 2251–2261. 23919167 10.1002/ece3.624 PMC3728962

[ece35099-bib-0057] Vatanparast, M. , Takayama, K. , Sousa, M. S. , Tateishi, Y. , & Kajita, T. (2011). Origin of Hawaiian endemic species of *Canavalia* (Fabaceae) from sea‐dispersed species revealed by chloroplast and nuclear DNA sequences. Journal of Japanese Botany, 86, 15–25.

[ece35099-bib-0058] Verdcourt, B. (1971). Phaseoleae In Milne‐Redhead E., & Polhill R. M. (Eds.), Flora of tropical East Africa, Leguminosae (part 4), Papilionoideae (2) (pp. 625–627). London, UK: Crown Agents for Overseas Governments.

[ece35099-bib-0059] Voris, H. K. (2000). Maps of Pleistocene sea levels in Southeast Asia: shorelines, river systems and time durations. Journal of Biogeography, 27, 1153–1167. 10.1046/j.1365-2699.2000.00489.x

[ece35099-bib-0060] Wee, A. K. S. , Takayama, K. , Chua, J. L. , Asakawa, T. , Meenakshisundaram, S. H. , Onrizal, A. B. , … Kajita, T. (2015). Genetic differentiation and phylogeography of partially sympatric species complex *Rhizophora* *mucronata* Lam. and *R. stylosa* Griff. using SSR markers. BMC Evolutionary Biology, 15, 57. 25888261 10.1186/s12862-015-0331-3 PMC4389924

[ece35099-bib-0061] Wee, A. , Teo, J. , Chua, J. , Takayama, K. , Asakawa, T. , Meenakshisundaram, S. , … Webb, E. (2017). Vicariance and oceanic barriers drive contemporary genetic structure of widespread mangrove species *Sonneratia* *alba* J. Sm in the Indo‐West Pacific. Forests, 8, 483 10.3390/f8120483

