# Supplementary material for: The presence of a cryptic barrier in the West Pacific Ocean suggests the effect of glacial climate changes on a widespread sea‐dispersed plant, Vigna marina (Fabaceae)
Source: Ecol Evol. 2019 Jul 4;9(15):8429–40. doi: 10.1002/ece3.5099 (PMC6686344; doi:10.1002/ece3.5099)

Figure S1

The prior and posterior distributions for each parameter of scenario2 in ABC1 obtained using DIYABC. The Y-axis represents the probability densities of priors and posteriors.

Figure S2

Principal Component Analysis (PCA) of scenario2 in ABC1 obtained by DIYABC.

Figure S3

The prior and posterior distributions for each parameter of scenario2 in ABC2 obtained using DIYABC. The Y-axis represents the probability densities of priors and posteriors.


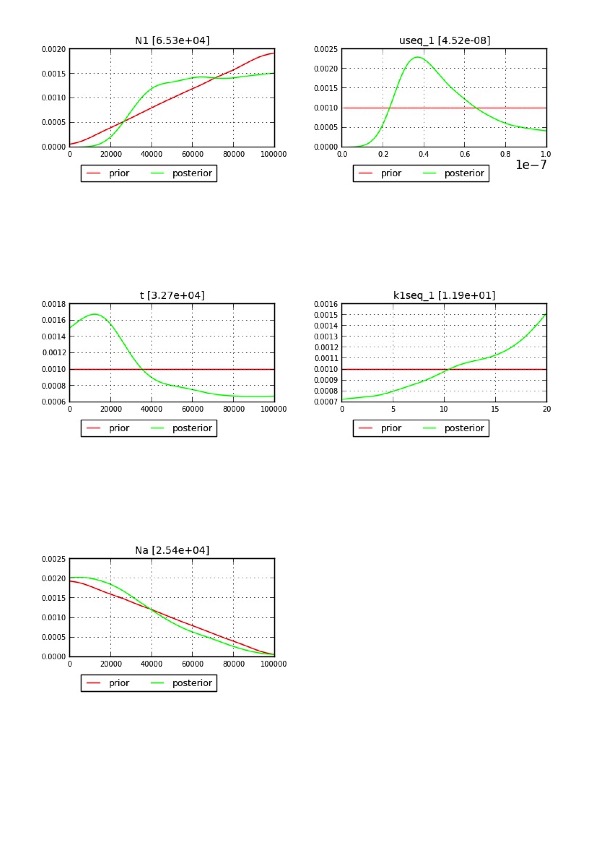


Figure S4

Principal Component Analysis (PCA) of scenario2 in ABC2 obtained by DIYABC.

Figure S5

Population NJ tree based on DA genetic distance. The values shown are the percentages of 1,000 bootstrap replicates supporting the respective nodes (only values exceeding 50 are shown).


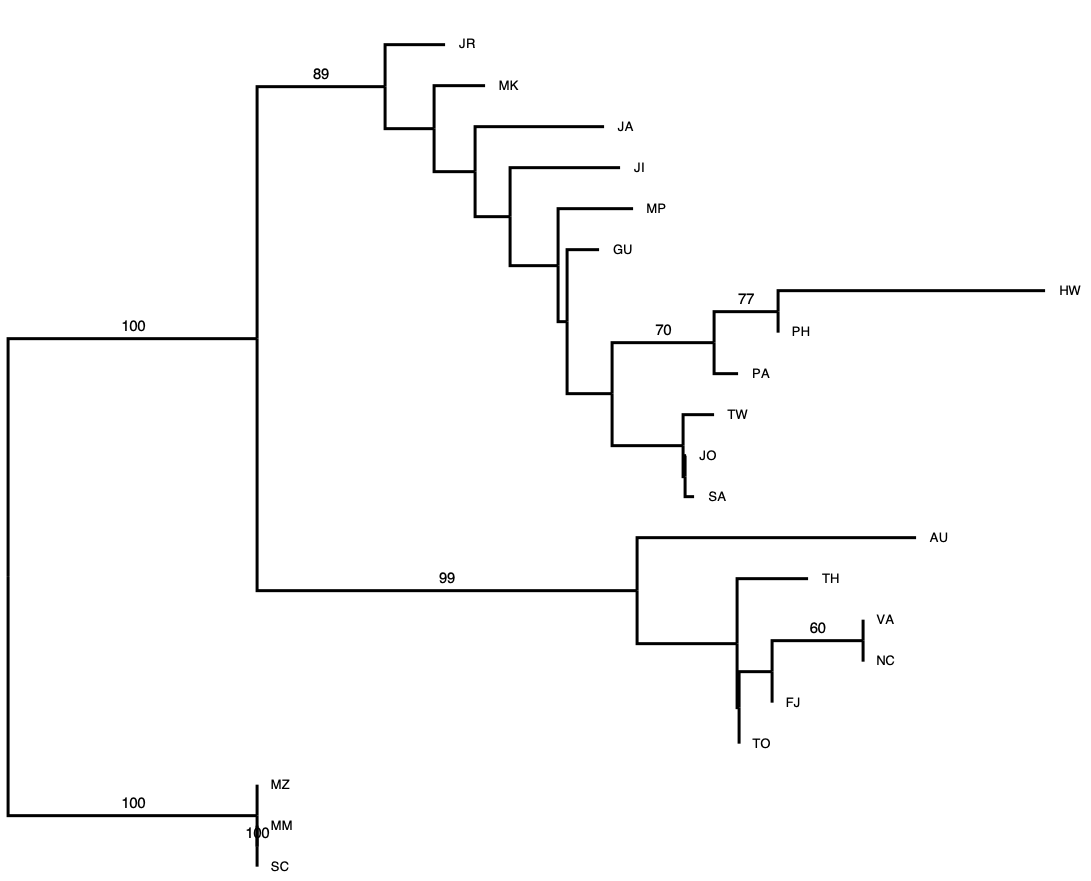

Supplement: Supplementary file 1 [file ECE3-9-8429-s001.docx]
