# Supplementary material for: The presence of a cryptic barrier in the West Pacific Ocean suggests the effect of glacial climate changes on a widespread sea‐dispersed plant, Vigna marina (Fabaceae)
Source: Ecol Evol. 2019 Jul 4;9(15):8429–40. doi: 10.1002/ece3.5099 (PMC6686344; doi:10.1002/ece3.5099)
Supplement: Supplementary file 2 [file ECE3-9-8429-s002.docx]

| Table S1 |  |  |  |  |  |  |  |  |
| --- | --- | --- | --- | --- | --- | --- | --- | --- |
| Localities of samples of *Vigna marina* and *Vigna hosei*. *N* indicates number of samples using data analysis. | | | | | | | | |
|  |  |  |  |  |  |  |  |  |
| Taxa | Oceanic region | Locality | | Voucher | Longitude | Latitude |  | *N* |
| *Vigna marina* (Burm.) Merr. | Pacific Ocean | Japan | Asani, Amami I. | YTM1211251 | E129.478272 | N28.399800 |  | 4 |
|  |  |  | Inno-jyouhuta, Tokunoshima I. | YTM1211231 | E128.887714 | N27.807271 |  | 4 |
|  |  |  | Inutabu-minami, Tokunoshima I. | YTM1211232 | E128.892961 | N27.712178 |  | 2 |
|  |  |  | Hentona, Ryukyu I. | TK13092801 | E128.182527 | N26.748633 |  | 5 |
|  |  |  | Awa, Ryukyu I. | TK13092801 | E128.293897 | N26.716059 |  | 10 |
|  |  |  | Hahajima I., Ogasawara | TK13100404 | E142.142472 | N26.698111 |  | 16 |
|  |  |  | Hyakuna, Ryukyu I. | TK13092701 | E127.796417 | N26.139833 |  | 16 |
|  |  |  | Sonai, Iriomote I. | TK01012904 | E123.749982 | N24.370652 |  | 2 |
|  |  |  | Haemidanohama beach, Iriomote I. | TK01013009 | E123.831304 | N24.272279 |  | 4 |
|  |  | Taiwan | Jialeshue, Pingtung | TK10032402 | E120.876635 | N22.009236 |  | 11 |
|  |  | U.S.A. | Oahu I., Hawaii | TK11053002 | W158.204428 | N21.581600 |  | 7 |
|  |  |  | Nimitiz beach, Guam I. | YT59544 | E144.649658 | N13.364680 |  | 4 |
|  |  |  | Talofofo bay, Guam I. | YT59535 | E144.760000 | N13.337778 |  | 9 |
|  |  |  | Bear Rock, Guam I. | YT59548 | E144.740167 | N13.266222 |  | 13 |
|  |  | Philippine | Tangalan, Panay I. | TK11062504 | E122.225980 | N11.794470 |  | 12 |
|  |  | Palau | Namai bay, Babeldaob I. | YT60931 | E134.640472 | N7.670611 |  | 9 |
|  |  |  | Choll, Babeldaob I. | YT60932 | E134.617278 | N7.522417 |  | 5 |
|  |  | Micronesia | Dekehtik causeway, Pohnpei I. | TK13121701 | E158.205440 | N6.976270 |  | 30 |
|  |  |  | Blue hole, Kosrae I. | TK12121702 | E163.020939 | N5.346019 |  | 31 |
|  |  | Samoa | Lalomanu, Upol I. | TK02102707 | W171.450240 | S14.04597 |  | 13 |
|  |  | Australia | Cape Tribulation, Queensland | TK04121304 | E145.466567 | S16.088507 |  | 1 |
|  |  | French Polynesia | Faauanu, Tahiti I. | KT14022508 | W149.376000 | S17.537806 |  | 16 |
|  |  |  | Vaioopu river, Tahiti I. | KT07100104 | W149.549556 | S17.750472 |  | 2 |
|  |  |  | Vaitepiha river, Tahiti I. | KT07100113 | W149.165139 | S17.751651 |  | 5 |
|  |  |  | Atiti, Tahiti I. | KT14022504 | W149.385389 | S17.759972 |  | 11 |
|  |  | Vanuatu | South of Erkao, Efate I. | TK10080211 | E168.443333 | S17.816667 |  | 5 |
|  |  | Fiji | Suva, Viti Levu I. | TK09012604 | E178.445883 | S18.156033 |  | 1 |
|  |  |  | Baravi, Viti Levu I. | TK09012803 | E177.571050 | S18.179150 |  | 11 |
|  |  | Tonga | Fufangalupe beach, Tongatapu I. | TK02102404 | W175.17111 | S21.256610 |  | 11 |
|  |  | New Caledonia | Yate, South Province | KT07092806 | E166.951461 | S22.166780 |  | 14 |
|  | Indian Ocean | Myanmar | Mein ma-hla-kyun, Ayeyarwady | TK11100905 | E95.260728 | N15.973264 |  | 5 |
|  |  | Myanmar | Twabaing, Ayeyarwady | TK11100806 | E95.359547 | N15.950594 |  | 5 |
|  |  | Seychelles | Baie Bean Vallon, Praslin I. | KT05090904 | E55.431250 | S4.615111 |  | 11 |
|  |  | Mozambique | Costa del sol, Maputo | TK10122804 | E32.651420 | S25.911630 |  | 3 |
|  |  |  |  |  |  |  | Total | 308 |

Table S2

Prior distributions of the parameters used in DIYABC.

| *ABC1* |  |  |
| --- | --- | --- |
| Parameter | Minimum | Maximum |
| *Effective population size* |  |  |
| N1 | 10 | 100000 |
| N2 | 10 | 100000 |
| N3 | 10 | 100000 |
| Na | 10 | 100000 |
| *Time scale in generations* |  |  |
| t1 | 10 | 100000 |
| t2 | 10 | 100000 |
| *Mutation model* |  |  |
| Mutation model | Hasegawa-Kishino-Yano (1985) | |
| Mean mutation rate | 1×10^-9^ | 1×10^-7^ |
| Individual locus mutation rate | 1×10^-9^ | 1×10^-6^ |
| Mean coefficient k C/T | 5×10^-2^ | 2×10^1^ |
| Individual locus coefficient k C/T | 5×10^-3^ | 2×10^2^ |
|  |  |  |
| *ABC2* |  |  |
| Parameter | Minimum | Maximum |
| *Effective population size* |  |  |
| N1 | 10 | 100000 |
| Na | 10 | 100000 |
| *Time scale in generations* |  |  |
| t | 10 | 100000 |
| *Mutation model* |  |  |
| Mutation model | Hasegawa-Kishino-Yano (1985) | |
| Mean mutation rate | 1×10^-9^ | 1×10^-7^ |
| Individual locus mutation rate | 1×10^-9^ | 1×10^-6^ |
| Mean coefficient k C/T | 5×10^-2^ | 2×10^1^ |
| Individual locus coefficient k C/T | 5×10^-3^ | 2×102 |

Table S3

Demographic parameters obtained by DIYABC.

| *ABC1* Scenario 2 |  |  |  |  |  |  |  |  |  |
| --- | --- | --- | --- | --- | --- | --- | --- | --- | --- |
|  |  |  |  | quantile | | | | | |
| Parameter | mean | median | mode | 2.50% | 5% | 25% | 75% | 95% | 97.50% |
| N1 | 84100.00 | 87200.00 | 98200.00 | 52600.00 | 58600.00 | 77200.00 | 94000.00 | 98800.00 | 99400.00 |
| N2 | 23400.00 | 18100.00 | 12200.00 | 3640.00 | 4730.00 | 10600.00 | 30400.00 | 62900.00 | 76600.00 |
| N3 | 6010.00 | 3460.00 | 1400.00 | 271.00 | 461.00 | 1660.00 | 7150.00 | 19900.00 | 27900.00 |
| Na | 37400.00 | 31700.00 | 5190.00 | 1230.00 | 2670.00 | 13800.00 | 57800.00 | 89900.00 | 94600.00 |
| t1 | 9170.00 | 6740.00 | 4040.00 | 1510.00 | 1970.00 | 4220.00 | 11200.00 | 24800.00 | 31800.00 |
| t2 | 42300.00 | 38200.00 | 26400.00 | 8510.00 | 11000.00 | 23800.00 | 57800.00 | 87500.00 | 93700.00 |
| Mean mutation rate | 2.92×10^-8^ | 2.74×10^-8^ | 2.80×10^-8^ | 1.51×10^-8^ | 1.67×10^-8^ | 2.23×10^-8^ | 3.37×10^-8^ | 4.81×10^-8^ | 5.50×10^-8^ |
| Mean coefficient k C/T | 1.15×10^1^ | 1.22×10^1^ | 2.00×10^1^ | 8.15×10^-1^ | 1.52×10^0^ | 6.75×10^0^ | 1.67×10^1^ | 1.97×10^1^ | 2.00×10^1^ |
|  |  |  |  |  |  |  |  |  |  |
| *ABC2* Scenario 2 |  |  |  |  |  |  |  |  |  |
|  |  |  |  | quantile | | | | | |
| Parameter | mean | median | mode | 2.50% | 5% | 25% | 75% | 95% | 97.50% |
| N1 | 64900.00 | 65300.00 | 97200.00 | 26900.00 | 30600.00 | 47300.00 | 83300.00 | 96900.00 | 98400.00 |
| Na | 29800.00 | 25400.00 | 6240.00 | 1290.00 | 2570.00 | 12200.00 | 43400.00 | 72000.00 | 79900.00 |
| t | 40200.00 | 32700.00 | 14000.00 | 2320.00 | 4250.00 | 15300.00 | 63200.00 | 92600.00 | 96500.00 |
| Mean mutation rate | 4.96×10^-8^ | 4.52×10^-8^ | 3.60×10^-8^ | 2.32×10^-8^ | 2.55×10^-8^ | 3.46×10^-8^ | 6.15×10^-8^ | 8.86×10^-8^ | 9.39×10^-8^ |
| Mean coefficient k C/T | 1.14×10^1^ | 1.19×10^1^ | 2.00×10^1^ | 7.81×10^-1^ | 1.45×10^0^ | 6.57×10^0^ | 1.64×10^1^ | 1.96×10^1^ | 1.99×10^1^ |
|  |  |  |  |  |  |  |  |  |  |

Table S4

Comparison of summary statistics for the observed data set and posterior simulated data sets.

| *ABC1* |  |  |
| --- | --- | --- |
| Summary statistics | observed value | *P*-value (simulated < observed) |
| Number of haplotypes in Pop1 | 26.000 | 0.8715 |
| Number of haplotypes in Pop2 | 5.000 | 0.3985 |
| Number of haplotypes in Pop3 | 1.000 | 0.2335 |
| Number of segregating sites in Pop1 | 29.000 | 0.6255 |
| Number of segregating sites in Pop2 | 11.000 | 0.6330 |
| Number of segregating sites in Pop3 | 0.000 | 0.2335 |
| Mean of pairwise differences in Pop1 | 3.009 | 0.3855 |
| Mean of pairwise differences in Pop2 | 2.151 | 0.5365 |
| Mean of pairwise differences in Pop3 | 0.000 | 0.2335 |
| Variance of pairwise differences in Pop1 | 7.535 | 0.5710 |
| Variance of pairwise differences in Pop2 | 14.085 | 0.8480 |
| Variance of pairwise differences in Pop3 | 0.000 | 0.2335 |
| Tajima's D in Pop1 | -1.087 | 0.1950 |
| Tajima's D in Pop2 | -0.106 | 0.3370 |
| Tajima's D in Pop3 | 0.000 | 0.5195 |
| Private segregating sites in Pop1 | 19.000 | 0.3750 |
| Private segregating sites in Pop2 | 1.000 | 0.0995 |
| Private segregating sites in Pop3 | 0.000 | 0.2370 |
| Mean of numbers of the rarest nucleotide at segregating sites in Pop1 | 13.296 | 0.2165 |
| Mean of numbers of the rarest nucleotide at segregating sites in Pop2 | 8.546 | 0.3175 |
| Mean of numbers of the rarest nucleotide at segregating sites in Pop3 | 0.000 | 0.2335 |
| Variance of numbers of the rarest nucleotide at segregating sites in Pop1 | 214.357 | 0.1380 |
| Variance of numbers of the rarest nucleotide at segregating sites in Pop2 | 12.793 | 0.2150 |
| Variance of numbers of the rarest nucleotide at segregating sites in Pop3 | 0.000 | 0.3845 |
| Number of haplotypes (Pop1 & Pop2) | 30.000 | 0.8165 |
| Number of haplotypes (Pop1 & Pop3) | 27.000 | 0.8300 |
| Number of haplotypes (Pop2 & Pop3) | 6.000 | 0.2920 |
| Number of segregating sites (Pop1 & Pop2) | 30.000 | 0.4415 |
| Number of segregating sites (Pop1 & Pop3) | 30.000 | 0.4870 |
| Number of segregating sites (Pop2 & Pop3) | 13.000 | 0.4370 |
| Mean of pairwise differences (W) (Pop1 & Pop2) | 2.906 | 0.3800 |
| Mean of pairwise differences (W) (Pop1 & Pop3) | 2.971 | 0.3835 |
| Mean of pairwise differences (W) (Pop2 & Pop3) | 1.966 | 0.5250 |
| Mean of pairwise differences (B) (Pop1 & Pop2) | 5.807 | 0.7320 |
| Mean of pairwise differences (B) (Pop1 & Pop3) | 4.759 | 0.3080 |
| Mean of pairwise differences (B) (Pop2 & Pop3) | 5.416 | 0.3820 |
| *F*_ST_ (Pop1 & Pop2) | 0.500 | 0.9245 |
| *F*_ST_ (Pop1 & Pop3) | 0.376 | 0.4500 |
| *F*_ST_ (Pop2 & Pop3) | 0.637 | 0.4480 |
|  |  |  |
| *ABC2* |  |  |
| Summary statistics | observed value | *P*-value (simulated < observed) |
| Number of haplotypes | 26.000 | 0.7515 |
| Number of segregating sites | 29.000 | 0.4235 |
| Mean of pairwise differences | 3.009 | 0.2895 |
| Variance of pairwise differences | 7.535 | 0.4760 |
| Tajima's D | -1.087 | 0.2285 |
| Private segregating sites | 29.000 | 0.4235 |
| Mean of numbers of the rarest nucleotide at segregating sites | 13.296 | 0.2380 |
| Variance of numbers of the rarest nucleotide at segregating sites | 214.357 | 0.1480 |
| **P* < 0.05, ***P* < 0.01 |  |  |
